# News and Coming Events

**Published:** 1909-08-14

**Authors:** 


### News and Coming Events.

Mr. John Dyer has been elected Chairman of the Swansea Hospital Board.

The proceeds of a collection on behalf of the poor of West Molesey and the East and West Molesey Cottage Hospital, made at Hurst Park recently, amounted to £53 18s. (including £17 8s. from those in the public stand).

The new Cottage Hospital at Torrington, erected in memory of the late Hon. Mark Rolle, was opened last week. The building can acommodate ten patients, and has been fitted up with improved operating and consulting rooms.

Sir Malcolm Morris and Dr. Newsholme, M.O.H. to the Local Government Board, have been appointed Government delegates to the International Congress on Leprosy to be held at Bergen next week. All the various colonies are represented at the Congress.

Mr. W. M. Mollison, M.C., F.R.C.S., has been appointed Arthur Durham Travelling Student at Guy's Hospital. Mr. Mollison, who is one of the assistant surgeons at the Evelina Hospital, has held the usual students' appointments, and is at present one of the demonstrators of anatomy at Guy's Hospital Medical School.

On November 2 next the National Hospital for the Paralysed and Epileptic, Bloomsbury, will celebrate its jubilee, and two days later the King will visit the institution for the purpose of inaugurating the various alterations now in progress. In connection with the jubilee the Princess of Wales has promised to visit the Hospital on October 9 to receive purses of not less than ten pounds each for the fund. On November 2 the Duchess of Albany will attend to receive purses of smaller amounts, and on the same day the Archbishop of Canterbury will preach at a special thanksgiving service in the hospital chapel.

At a meeting of the English Committee of the Sixteenth International Congress of Medicine, held at the rooms of the Medical Society of London on July 22, 1909, Dr. F. W. Pavy, F.R.S., President, in the chair, it was proposed by Sir William Sinclair, seconded by Mr. Eliot Creasy, that the members of the National Committee be circularised as to the advisability of asking H.M.'s Government to invite the Seventeenth International Congress of Medicine to meet in this country. A report was made upon the appointment of official delegates at the Budapest Congress. Members of the Congress who desire to travel with the Royal Society of Medicine party should communicate at once with Mr. J. Y. W. MacAlister, at 20 Hanover Square, London, W.

Dr. Arthur Foxwell, who died in the Warneford Hospital, Leamington, on August 4, at the age of 56, as the result of a cycle accident on August 1, was born at Shepton Mallet in 1853 and was educated at Queen's College, Taunton, and at Cambridge. After studying at St. Thomas's Hospital and at the General Hospital of Vienna, he became successively Pathologist and Assistant Physician at the Birmingham General Hospital, and was for a time examiner in medicine for Cambridge University. He edited the *Birmingham Medical Review*, and was librarian of the Birmingham Medical Institute. As Senior Physician at the Queen's Hospital and Professor of Therapeutics in the University of Birmingham he was well known and highly esteemed by his professional colleagues and all who knew him.

A Parliamentary Paper just published shows that on June 26 the aggregate number of paupers on the relief lists amounted to 784,434 persons (or 22.2 per 1,000 of the population), a decrease of 6.7 per cent. in the quarter. Compared with the position in the corresponding period of 1908 the figures show a substantial increase in the number of persons relieved in the present year. The rate of increase during April ranged between 1.9 and 3.8 per cent., but it fell considerably during May (ranging between 0.9 and 1.3). A slight increase occurred during June, and on the last Saturday in the month the numbers relieved were 2.0 per cent. in excess of those recorded on June 27, 1908. The rate of pauperism to population shows a corresponding increase, standing at 22.2 per 1,000 of the population at the end of June, as compared with 22.0 per 1,000 on the corresponding day in 1908.

At the last meeting of the board of governors of the Battersea General Hospital the following resolution was carried unanimously :—"That in respect of the grant of £97 10s. voted by the Council of the Metropolitan Hospital Sunday Fund, the board of the National Anti-Vivisection Hospital and Battersea General Hospital acknowledges with grateful thanks the action of those friendly to the Anti-Vivisection Hospital in voting and carrying the vote of the council of the Metropolitan Hospital Sunday Fund, and appreciates their efforts, upon which it sets a very high value. The board, however, cannot overlook the fact of the deeply injurious remarks made by certain members of the council and distribution committee, not only as regards the system of the Anti-Vivisection Hospital, but also the efficiency of its treatment. To emphasise the board's denial of those offensive assertions, which have not the slightest foundation in fact, it resolved not to accept the grant named, or any grant, until it can be voted in an impartial and dignified manner."

The time has apparently come for an effort to be made to gather together in one nucleus the various units of progressive and experimental thought which to-day constitute the psychical, spiritistic, and spiritual interests of society. It is with this object in view that an International Club for Psychical Research has just been started in London. Besides the usual accompaniments of a club—*table d'hôte*, general and private reception, reading and writing rooms, residential facilities for country and foreign members—a large and complete library of the literature of the psychical and all "occult" sciences will be supplied for the use of members. It is hoped to secure as President a man of scientific qualifications and authority, under whose direction the committee will arrange for lectures to be given at short and regular intervals. A bulletin of the proceedings will be published regularly for free circulation among members; systematic arrangements will be made for the development of promising mediums, for holding long series of experimental séances on the premises of the club (members only to participate). Appliances and apparatus for the study of the physical effects of mediumship, photographic facilities, etc., will be supplied for the exclusive use of members. Suitable premises for the Club House have been secured close to Piccadilly and Pall Mall, in the heart of Clubland, and the opening ceremony will take place early in October. Further particulars can be obtained of Mr. Wynan Hope, *Annals of Psychical Science*, 110 St. Martin's Lane, W.C., or of Mr. Dudley Wright, Editor of the *Annals*.

